# A Review of DNA Vaccines Against Influenza

**DOI:** 10.3389/fimmu.2018.01568

**Published:** 2018-07-09

**Authors:** Leo Yi Yang Lee, Leonard Izzard, Aeron C. Hurt

**Affiliations:** World Health Organisation Collaborating Centre for Reference and Research on Influenza at the Peter Doherty Institute, Melbourne, VIC, Australia

**Keywords:** DNA vaccine, influenza, adjuvant, hemagglutinin, immunization

## Abstract

The challenges of effective vaccination against influenza are gaining more mainstream attention, as recent influenza seasons have reported low efficacy in annual vaccination programs worldwide. Combined with the potential emergence of novel influenza viruses resulting in a pandemic, the need for effective alternatives to egg-produced conventional vaccines has been made increasingly clear. DNA vaccines against influenza have been in development since the 1990s, but the initial excitement over success in murine model trials has been tempered by comparatively poor performance in larger animal models. In the intervening years, much progress has been made to refine the DNA vaccine platform—the rational design of antigens and expression vectors, the development of novel vaccine adjuvants, and the employment of innovative gene delivery methods. This review discusses how these advances have been applied in recent efforts to develop an effective influenza DNA vaccine.

## Introduction

Seasonal influenza epidemics continue to challenge public health systems worldwide, causing 3–5 million cases of severe respiratory disease and 290–650 thousand deaths annually (1). Despite annual updates to the seasonal vaccine, in 2017 overall vaccine effectiveness for Australia was estimated to be only 33% (2), and interim estimates from the United States were similarly low for the 2017–2018 influenza seasons (36%) (3). In addition, current seasonal vaccines provide little or no protection against novel pandemic viruses of animal origin (4). Consequently, research efforts have increased to improve seasonal vaccines and develop new vaccine platforms to achieve better protection against both seasonal and potentially pandemic influenza A viruses.

**Figure 1:**
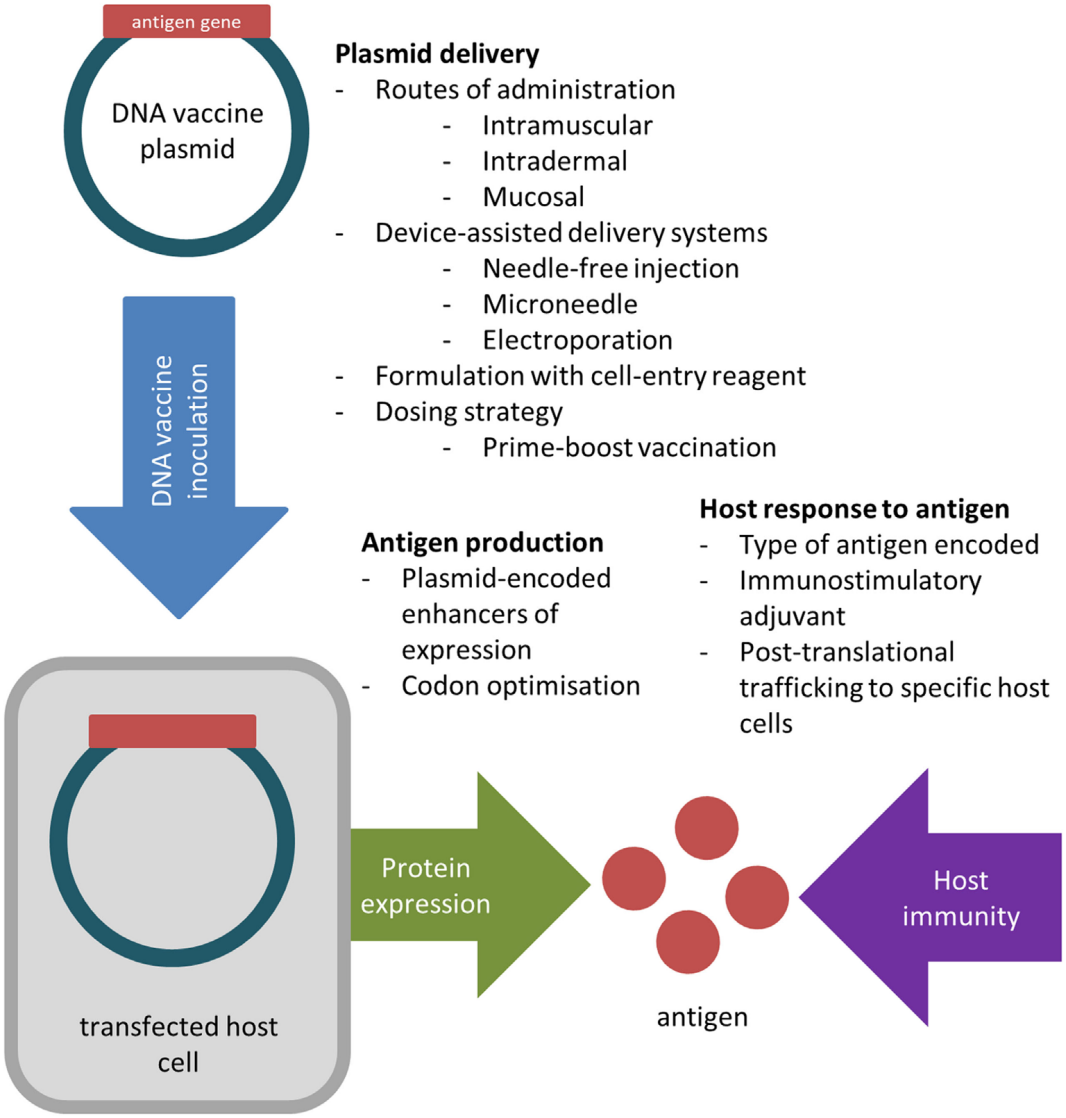
Key factors considered in the design, formulation, and inoculation of DNA vaccines to improve *in vivo* transfection efficiency and antigen immunogenicity.

DNA vaccines possess numerous properties ideal for influenza control and have been trialled for a range of diseases, including viral and bacterial infections, and some cancers (5–7). Whilst inactivated influenza vaccines (IIVs) largely rely on antibody production to achieve effective protection (8), DNA vaccines can efficiently engage both humoral and cell-mediated immune responses (9). Their production does not require the growth of live virus and can be rapidly upscaled in response to emerging pandemic influenza (10, 11). Despite these advantages, promising immunogenic responses achieved in small animal models, predominantly mice, are rarely replicated in larger animals (12, 13). Murine model data are based on immune responses in highly inbred animals to mouse-adapted influenza viruses—an unreliable comparison to vaccination in the outbred human population against circulating influenza viruses (14, 15). Larger animal models susceptible to human influenza virus provide more relevant data—ferrets exhibit clinical signs, lung pathology, and transmission similar to humans (16, 17), whist human-like immune responses to influenza in cynomolgus macaques are good predictors of vaccine efficacy in humans (15, 18). As such, achieving sufficient immunogenicity in larger animals has required the development of potent delivery systems and adjuvants (19, 20). This review summarises innovations in the design, formulation, and delivery of DNA vaccines against influenza, and the major obstacles impeding their implementation (Figure 1).

## Influenza Vaccines: Production and Mechanisms of Protection

Inactivated influenza vaccines and live attenuated influenza vaccines (LAIV) are the most widely used forms of influenza vaccine, and are generated by harvesting viruses grown in embryonated hen’s eggs (21). The delivery of viral antigens derived from this process induces the production of antigen-specific antibodies, particularly against the haemagglutinin (HA) surface glycoprotein, to protect against future infections (22). However, egg-based vaccine production is time-consuming and resource-intensive, and manufacturing delays have previously caused severe vaccine shortages (23, 24). The overall vaccine effectiveness against seasonal influenza ranges from 40 to 60% during typical seasons, but is significantly reduced when antigenic mismatch occurs (25, 26). Furthermore, antigenic mismatch can be exacerbated by mutations which allow vaccine viruses to grow in eggs, which may also alter antigenic sites (27).

DNA vaccines are able to avoid many issues associated with egg-based vaccine production by generating viral proteins within host cells. To create a DNA vaccine, an antigen-encoding gene is cloned into a non-replicative expression plasmid, which is delivered to the host by traditional vaccination routes (28). Host cells which take up the plasmid express the vaccine antigen which can be presented to immune cells *via* the major histocompatibility complex (MHC) pathways. CD4+ T helper cell activation following MHC class II presentation of secreted DNA vaccine protein is critical for the production of antigen-specific antibodies (29), whist CD8+ T cell immunity, important for viral clearance, is predominantly activated by endogenously expressed antigens presented on MHC class I molecules (30).

## Designing Antigens for Influenza DNA Vaccines

The protection conferred by conventional IIV is based on the induction of HA-specific serum antibodies, which interfere with virus attachment to inhibit cell entry and limit infection (8, 31). Early mouse studies using DNA vaccines encoding H1 HA genes reported that protection against lethal homologous challenge correlated with increasing titres of HA-specific serum antibody (32, 33). As observed in LAIV, other correlates of immunity are less well defined, but the induction of local inflammation and cytotoxic T cell responses have been implicated as key mechanisms to enhance vaccine cross-reactivity and reduce the severity of infections (34, 35). As such, the nature of the host response to influenza DNA vaccination can be manipulated by the encoded antigen.

“Universal” influenza vaccines are being developed to induce broadly protective responses against drifted variant viruses and animal-origin strains that may result in a pandemic. Protection induced by evolutionarily stable influenza antigens is associated with viral clearance mediated by broadly reactive cytotoxic CD8+ T cells, reducing the severity of clinical disease (36). Candidate universal influenza vaccine targets include the nucleoprotein (NP), matrix proteins (M1 and M2), and the RNA-directed RNA polymerase catalytic subunit (PB1). Individual plasmids encoding NP (37) and M2 (38) have each been reported to decrease viral load and enhance survival against lethal heterologous challenge viruses in BALB/c mice. Combined immunisation with matrix protein, NP, and PB1 plasmids has been reported to induce protection against heterologous challenge in mice (39), pigs (40), ferrets (41), and macaques (42). Chimeric protein antigens designed to increase the breadth of host responses can be delivered by DNA vaccines. Plasmid-encoded fusion proteins of H1N1 HA and the conserved M2-ectodomain improved the cross-reactivity of antibody responses to drifted H1N1 viruses compared to a plasmid encoding HA alone in mice (43).

Attempts to create HA-based universal influenza vaccines have targeted the conserved stem region of the HA protein (31). Mice vaccinated with plasmids encoding a PR8 “headless HA” antigen developed serum antibody responses to a greater range of influenza viruses than wild-type HA DNA-vaccinated animals (44). The expression of consensus HA sequences has also increased the cross-reactivity of antibody responses (45–47). Chen et al. (48) constructed a plasmid encoding a consensus H5 HA generated from 467 HA sequences, which induced protection against a wide spectrum of lethal H5N1 reassortant challenge viruses in mice. Broadly reactive responses have also been induced using polyvalent formulations similar to currently available trivalent and quadrivalent IIVs (26). Huber et al. (49) generated cross-reactive antibodies against multiple H3 drift variants in mice by vaccinating with three different H3-expressing plasmids. Rao et al. (50) achieved similar success against several variant H5N1 viruses in chickens using vaccines containing up to 10 different H5 HA plasmids.

Efficient antigen expression *in situ* is a key factor for DNA vaccine effectiveness which can be modulated by altering the antigen coding sequence. Encoding antigens using codons optimised for expressing within the host species is a commonly used strategy to enhance influenza DNA vaccine expression (51–53). Jiang et al. (54) used a lethal H5N1 challenge model in chickens to compare the protective efficacy of DNA vaccines encoding either the wild-type HA or HA codon-optimised for chickens. Chickens receiving the codon-optimised HA plasmid demonstrated up to fourfold increases in antibody titre compared to animals inoculated with wild-type HA plasmids, resulting in greater survival rates during viral challenge.

DNA vaccine antigen design can direct the post-translational trafficking of expressed proteins to influence the development of host immunity. The human tissue plasminogen activator leader sequence promotes high levels of protein secretion and has improved antibody responses to an H5 HA DNA vaccine in rabbits (55). Grodeland et al. (29) encoded DNA vaccine antigens consisting of H1 HA linked to MHC class II-targeting units which enhanced its delivery to antigen-presenting cells (APCs). Ferrets and pigs vaccinated with plasmids expressing the targeted H1 fusion protein generated significant antibody titres, whereas H1 DNA alone failed to cause seroconversion. A similar DNA vaccine strategy expressing APC-targeted H7 fusion proteins was found to improve anti-HA serum antibody and cytotoxic T cell responses to highly pathogenic avian influenza (56).

## DNA Vaccine Delivery Platforms

For influenza DNA vaccines, the route of administration is critical to vaccine effectiveness as it dictates the cell types that will be transfected. DNA vaccines were initially tested in the murine model using intramuscular injection of naked plasmids to produce antigens in passively transfected myocytes (muscle cells) (57). This method relies on the influx of leucocytes following local inflammation to expose the immune system to DNA vaccine antigens (58). Outside of the murine model, effective intramuscular administration of plasmids depends on adjuvants and delivery systems to achieve sufficient immunogenicity (59). More recently, cutaneous delivery has become a highly desirable route for DNA vaccines, as the epidermis is abundant in Langerhans cells, which can efficiently transport and present DNA vaccine-encoded antigens in the lymph node (58).

Alternative delivery devices have been developed to improve upon traditional needle and syringe inoculation for parenteral administration. In small animal models, the gene gun induces immune responses successfully with low doses of DNA by delivering gas-propelled plasmid-coated gold microparticles directly into epidermal cells (60, 61). Human clinical trials of influenza DNA vaccines have successfully employed the Biojector system (iHealthNet, GA, USA), which uses pressurised CO_2_ to transport a liquid inoculum to the intradermal or intramuscular layer (62–64). Recently, the development of patches composed of micron-length needles has enabled the dermal delivery of lyophilised DNA vaccine (65–67). HA DNA vaccination in mice using dry-coated microneedle patches were reported to induce antibody titres and T cell responses up to five times higher than an equivalent intramuscular dose (68).

Parenteral gene delivery has been further enhanced by electroporation, which temporarily increases the permeability of local cell membranes with electrical pulses (69). Its early use alongside intramuscular delivery required highly invasive electrodes associated with excessive inflammation and the production of lesions (70). Updated devices such as the CELLECTRA system (Inovio, PA, USA) can target the dermal and subcutaneous layers and are optimised to be minimally invasive for clinical use (71). Electroporation has been reported to enable influenza DNA vaccines to generate robust antibody titres and T cell responses in guinea pigs (72), swine (73), and macaques (47, 74).

The mucosa is an appealing site of inoculation for influenza vaccines as it is easily accessible and is the clinical site of entry for influenza viruses (75). Existing mucosal influenza vaccines such as FluMist (MedImmune, MD, USA) mediate protection through local mucosal inflammation and the production of secretory IgA (34, 76). The enrichment of dendritic cells and M cells at mucosal surfaces is ideal for the immune presentation of DNA vaccine antigens (77, 78). However, successful mucosal delivery of plasmids in large animal models requires specialised adjuvants or highly optimised delivery systems (79, 80). Torrieri-Dramard et al. (81) reported that an intranasal HA DNA vaccine failed to elicit detectable IgA titres unless the plasmid was complexed with a polyethylenimine nanocarrier. To induce detectable seroconversion in sheep, Rajapaksa et al. (82) used a novel acoustic nebuliser to produce aerosols of an optimal size to deliver HA plasmids to deep lung tissue.

## Adjuvants

The co-administration of adjuvants with influenza DNA vaccines is a common strategy to elicit adequate levels of protection *in vivo*. The mechanisms of action for licensed conventional adjuvants include the formation of antigen depot at the inoculation site, the activation of inflammatory pathways, and the recruitment of APCs (83).

The goal of adjuvant design is to increase the immune response to vaccine antigens, a critical hurdle in the DNA vaccine field. Mineral salts such as alum are widely used in human vaccines and have resulted in up to fivefold increase in HA DNA vaccine-induced antibody titres (84). Cytokine expression vectors exploit host signalling pathways to heighten immune stimulation (85). IL-6 is an important inflammatory mediator involved in B cell stimulation and the recruitment of leucocytes (86, 87). Co-administration of an IL-6-expressing plasmid in HA DNA-vaccinated mice has been reported to reduce the duration of influenza illness (88). Lee et al. (60) reported that only 50% of HA and NP DNA-vaccinated mice survived a lethal homologous challenge, whereas mice receiving the additional IL-6-expressing plasmid were fully protected. The cytokine activity of high mobility group box 1 protein has been shown to increase the survival of mice vaccinated with NP DNA in a homologous viral challenge, and has been found to enhance antibody production induced by HA DNA vaccines by twofold (89). Cytokine adjuvants have also demonstrated effectiveness in large animal models such as macaques, where the use of adjuvant plasmids encoding GM-CSF, a potent immune cell proliferation and differentiation factor, resulted in up to fivefold increases in the serum antibody titre compared to a HA DNA vaccine delivered alone (90).

Adjuvant compounds developed as delivery reagents aim to improve the transfection efficiency of DNA vaccine plasmids. The efficiency of the cellular uptake of DNA vaccines is determined by cell membrane permeability and the susceptibility of foreign DNA to host enzymes. It is estimated that only 1% of a naked plasmid inoculation is able to reach the nuclei of target cells for protein expression—most plasmids remain in the extracellular space to be cleared by host processes (91). Synthetic nanocarriers form structures that protect DNA from host enzymes and facilitate its entry through the cell membrane lipid bilayer (85). Cationic lipids form vesicles known as liposomes, which interact electrostatically with negatively charged DNA to form lipoplexes that efficiently enter host cells through endocytosis (92). Vaxfectin (Vical, San Diego) is a cationic lipid-based system that has boosted influenza DNA vaccine immunogenicity in numerous large animal models (93–95). Other nanoparticle-forming polymers have been reported to enhance influenza DNA vaccine formulation including poly(lactic-co-glycolic) acid (96, 97), chitosan (98), and polyethylenimine (81).

## Prime-Boost Strategies

The administration of novel vaccine types including adenovirus vectors (99, 100), subviral particles (101), and recombinant protein antigens (102) in combination with conventional influenza vaccines has been reported to enhance seroconversion and antibody cross-reactivity. Wang et al. (103) demonstrated that a primary HA DNA vaccine followed by a seasonal trivalent inactivated vaccine (TIV) boost induced significantly higher antibody titres compared to two doses of either DNA vaccine or TIV in rabbits. Similar results have reported using DNA vaccines to prime LAIV in ferrets (104) and recombinant HA-protein vaccine in chickens (105). However, human trials applying this strategy against circulating seasonal influenza failed to significantly improve seroconversion compared to TIV alone (106, 107).

Despite this, studies have indicated that DNA vaccines may have a clinical application in pandemic settings. Chang et al. (108) demonstrated that mice which had been pre-exposed to H1N1 were significantly protected from lethal H5N1 challenge after DNA vaccination with H5N1 NP- and M1-expressing plasmids. Given the commonality of H1N1 exposure amongst the public, this suggests DNA vaccines could be rapidly deployed to protect a large susceptible population against H5N1 outbreaks. During the 2009 pandemic, a Phase 1 human clinical trial was conducted using an A(H1N1)pdm09 DNA vaccine produced 2 months before the licensed monovalent inactivated vaccine (MIV) (64). Seroconversion was observed in 30% of recipients after three doses of DNA vaccine delivered by Biojector, and the response rate rose to 72% after a booster dose of MIV. Similar human trials of DNA prime-MIV boost vaccines against H5N1 (109) and H7N9 (110) have reported significant improvements in antibody responses compared to MIV alone, indicating that DNA vaccines can effectively prime the immune system against viruses where there is low pre-existing immunity in the population. These recent developments indicate the potential for further research into combined DNA vaccine/IIV strategies as viable control measures against novel influenza outbreaks.

## Future Prospects

After two decades of research, DNA vaccine technology is gaining maturity—several veterinary DNA vaccines are currently licensed for West Nile virus and melanoma (111), and significantly, the first commercial DNA vaccine against H5N1 in chickens has recently been conditionally approved by the USDA (112). In addition, ongoing large animal trials of DNA vaccines against other diseases such as against HIV (6, 113, 114), hepatitis (115, 116), and Zika virus (117, 118) offer valuable insights that can be applied to influenza DNA vaccine design. Promising approaches have arisen from the numerous studies evaluating different DNA vaccine formulations and delivery systems, but a strategy that consistently elicits protection against influenza in large animal models has not yet emerged. Successful plasmid delivery and the use of appropriate adjuvants remain key challenges that need to be addressed before influenza DNA vaccines become effective for human use.

## Author Contributions

All authors listed have made a substantial, direct, and intellectual contribution to the work and approved it for publication.

## Conflict of Interest Statement

The authors declare that they have no commercial or financial relationships that could be construed as a potential conflict of interest.
